# Perspectives on the Use of Ninjin'yoeito in Modern Medicine: A Review of Randomized Controlled Trials

**DOI:** 10.1155/2019/9590260

**Published:** 2019-09-02

**Authors:** Shin Takayama, Ryutaro Arita, Minoru Ohsawa, Akiko Kikuchi, Hiromichi Yasui, Toshiaki Makino, Yoshiharu Motoo, Tadashi Ishii

**Affiliations:** ^1^Department of Kampo Medicine, Tohoku University Hospital, 1-1 Seiryo-machi, Aoba Ward, Sendai, Miyagi 980-8574, Japan; ^2^Department of Kampo and Integrative Medicine, Tohoku University Graduate School of Medicine, 1-2 Seiryo-machi, Aoba Ward, Sendai, Miyagi, Japan; ^3^Department of Education and Support for Regional Medicine, Tohoku University Hospital, 1-1 Seiryo-machi, Aoba Ward, Sendai, Miyagi 980-8574, Japan; ^4^Japan Institute of TCM Research, 7-8 Kitahamacho, Yokkaichi, Mie 510-0091, Japan; ^5^Department of Pharmacognosy, Graduate School of Pharmaceutical Sciences, Nagoya City University, 3-1 Tanabe-Dori, Mizuho-ku, Nagoya 4678603, Japan; ^6^Department of Medical Oncology, Kanazawa Medical University, 1-1 Daigaku, Uchinada, Ishikawa 920-0293, Japan

## Abstract

**Background:**

Ninjin'yoeito (NYT), a traditional Japanese (Kampo) medicine that originates from China, has been used to treat qi and blood deficiency based on its original concept. Kampo medicine has been widely used to treat many conditions and disorders combined with western medicine or Kampo medicine alone in modern situation.

**Aims:**

We reviewed randomized controlled trials (RCTs) of NYT and discussed various standpoints regarding its use in modern situation.

**Methods:**

We searched PubMed, Cochrane Library, and Evidence Reports of Kampo Treatment (EKAT) for articles written in English, and Ichushi, J-Stage, and EKAT for those written in Japanese. Articles published before January 1, 2019, were retrieved using the keywords “ninjinyoeito” and “ninjin'yoeito,” and RCTs were selected from these extracted articles.

**Result:**

Of 734 articles, 13 were RCTs, 46 were non-RCTs or studies of other designs, 15 were case reports, and 36 were experimental studies using NYT. NYT was evaluated for its use as a treatment for cancer and related conditions, refractory blood diseases and conditions, and otorhinolaryngologic symptom in 13 RCTs. Based on the use of Kampo medicine in modern situation called as Yasui's classification, 10 of 13 RCTs were categorized as “the side effects of Western medicinal treatment are mitigated when combined with Kampo treatment” and the remaining 3 were categorized as “treatment effect of Kampo medicine is increased in combination with standard Western medicinal treatment.” *Conclusion*. Several studies demonstrated the efficacy of NYT in refractory diseases and other conditions, and the accompanied side effects of treatment with western medicine.

## 1. Introduction

Ninjin'yoeito (NYT), a traditional Japanese (Kampo) medicine that originates from China, is used to treat qi and blood deficiency pattern based on its original concept. NYT is especially used to treat spleen and lung qi deficiency pattern, and heart and liver blood deficiency pattern. NYT contains 12 crude drugs: Japanese Pharmacopoeia Seventeenth Edition (JP) Rehmannia Root, JP Japanese Angelica Root, JP Atractylodes Rhizome, JP Poria Sclerotium, JP Ginseng, JP Cinnamon Bark, JP Polygala Root, JP Peony Root, JP Citrus Unshiu Peel, JP Astragalus Root, JP Glycyrrhiza, and JP Schisandra Fruit [1]. Information on NYT is available from the website of STORK (http://mpdb.nibiohn.go.jp/stork/). Thus, NYT contains many physiologically active substances and has multifunctions in the improvement of symptoms and during recovery from diseases. This Kampo medicine is covered by the National Health Insurance system of Japan for declined constitution after recovery from diseases, fatigue and malaise, anorexia, perspiration during sleep, cold limbs, and anemia (Tsumura & Co., Japan, Kracie Pharmaceutical Ltd. Japan, and Ohsugi Pharmaceutical Co., Ltd Japan) [2], as well as for ill complexion, thin body, slight fever, chills, persistent cough, severe malaise, anorexia, mental disequilibrium, insomnia, night sweating, tendency to constipation, enhancement of physical strength after disease or childbirth, and delicate constitution (Kotaro Pharmaceutical Co., Ltd. Japan) [2]. Kampo medicine is widely used for the treatment of various conditions in modern medicine, and it has been applied to treat several conditions and disorders based on ancient concepts. Recently, NYT was applied to treat frailty in gastrointestinal, respiratory, and urinary functions in clinical settings. However, a review of its efficacy when used to treat several conditions or diseases has not been presented. Thus, in this study, we reviewed randomized controlled trials (RCTs) with NYT and discussed various standpoints regarding its use in modern medicine.

## 2. Methods

### 2.1. Literature Search

Using the keywords, “ninjinyoeito” or “ninjin'yoeito,” we conducted a database search of PubMed, Cochrane Library, and Evidence Reports of Kampo Treatment (EKAT) [3] for articles written in English, and Ichushi, J-Stage, and EKAT for those written in Japanese. The search was restricted to articles published before January 1, 2019. We also performed an additional “hand search” for recently published articles.

### 2.2. Selection Criteria

RCTs based on NYT were selected from a pool of research articles published in English or Japanese.

### 2.3. Data Extraction

Eligible articles were categorized by two independent researchers (ST and RA) who extracted and tabulated specific information from the articles. RCTs were classified based on their study design, number of subjects, intervention, control, and results. RCTs were then categorized into one of the following four types given in “Yasui's classification,” which depicts the recent prescription of Kampo medicine in Japan [4–10]: type 1, treatment is effective using Kampo medicine alone; type 2, the treatment effect is increased using Kampo medicine in combination with the standard western medicinal treatment; type 3, the side effects of western medicinal treatment are mitigated when combined with Kampo medicine; and type 4, situations where western medicine cannot be applied due to allergy, etc.

## 3. Results

**Figure 1:**
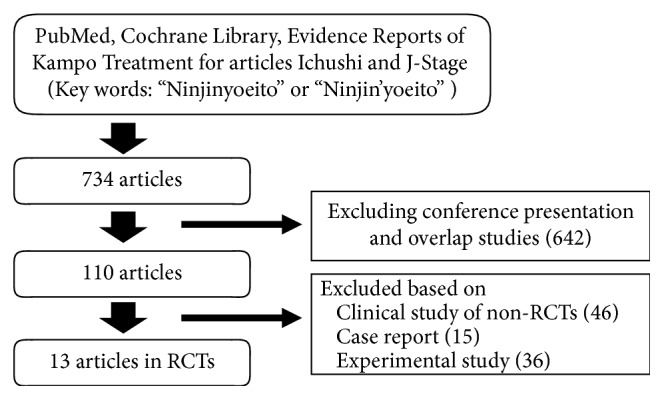
Flow chart of the review process.

The review process and number of eligible articles are shown in Figure 1. Of the 734 retrieved articles, there were 13 RCTs (Table 1), 46 clinical studies of non-RCTs or studies of other designs (17 articles on treatment for cancer and related conditions, 9 articles on blood diseases, 5 articles on otorhinolaryngology, 5 articles on collagen diseases, 5 articles on urogenital diseases, 5 articles in the other diseases), 15 case reports (7 articles on respiratory disorders, 3 articles on refractory blood diseases, 5 articles on the other diseases), and 36 experimental studies.

### 3.1. Randomized Controlled Trials (RCTs) of NYT (Table 1)

#### 3.1.1. Cancer Treatment and Related Condition

In 2004, Oda et al. reported the efficacy of NYT for bone-marrow suppression when chemotherapy was given to patients with gynecologic cancer. During the third cycle of chemotherapy, the duration of neutrophil count under 1,000/*μ*L was shorter in the NYT group than the control group, while total dose of granulocyte colony-stimulating factor (G-CSF) was significantly lower in the NYT group than the control group. Similarly, compared to the first cycle, the nadir hemoglobin level was significantly shorter in the second cycle in the NYT group, but not in the control group [11].

In 1995, Sugimachi reported the efficacy of NYT in reducing adverse effects and improving performance status (PS) in patients undergoing postoperative adjuvant chemotherapy (fluoropyrimidine anticancer drug) for gastric cancer. No significant difference was found between the groups regarding changes in body weight, PS, decline in white blood cell (WBC), red blood cell (RBC), and platelet (PLT) count, and subjective symptoms [12].

In 1995, Okawa et al. reported an improvement in subjective symptoms and leukopenia after NYT administration to patients undergoing radiotherapy for thoracoabdominal tumors. The proportion of patients with WBC count >3000/*μ*L at the end of treatment (weeks 4–8) was higher in the NYT group than the control group. Improvement in subjective symptoms and laboratory test results was also observed more frequently in the NYT group than the control [13].

In 1994, Hasegawa et al. reported the clinical usefulness of Kampo medicine including NYT for side effects in gynecologic cancer chemotherapy. Treatment with Kampo medicine did not significantly affect the decline in WBC, RBC, and PLT counts but tended to promote their reversal. Kampo medicine also reduced nephrotoxicity (i.e., normalized blood urea nitrogen level and reduced creatinine fluctuation), but subjective gastrointestinal symptoms did not improve [14].

In 1994, Yamamoto et al. reported the effects of NYT on subjective and objective symptoms and bone-marrow function during chemotherapy or radiotherapy in patients with genital cancer. An additional treatment with NYT significantly improved myelosuppressive symptoms, but not the subjective and objective symptoms associated with anticancer drug administration. It also improved anorexia and fatigue/malaise during radiotherapy. One patient had acute hepatitis with an unknown causal relationship with NYT [15].

In 1993, Ohara et al. reported the efficacy of Kampo medicine including NYT for cancer treatment with fluorouracil-related drugs. The subjective symptoms before and after treatment (nausea/vomiting, bowel movement abnormality, motivation, and fatigue/malaise) were significantly improved in the NYT group. Overall improvement was noted in 33.9% of patients in the NYT group and 14.3% of patients in the control group; a significant difference was found between the groups. Overall objective symptom improvement was noted in 39.3% of patients in the NYT group and 0.4% of patients in the control group; a significant difference was noted in the percentage of patients showing improvement between the two groups. The percentage of patients showing improvement in both subjective and objective symptoms was significantly greater in the NYT group than the control group but this was only for patients with gastric cancer. Side effects were present in 11.5% of patients. Efficacy, as determined by the attending doctor based on symptom improvement and side effects, was significantly greater than with conventional treatment [16].

In 1993, Mizuno et al. reported the efficacy of NYT for the relief of subjective symptoms and the improvement of activities related to daily living in patients after surgery for gynecologic cancer. Administering NYT could significantly reduce the subjective symptoms of physical strength, general fatigue, coldness in hand and foot, and night sweating compared to control. Only one case showed appetite loss and general fatigue, and these were categorized as side effects of treatment. The efficacy as determined by the attending doctor based on symptom improvement and side effects was significantly higher in the NYT group than the control [17].

Araki et al. reported the effect of NYT on immunostimulation and improved nutritional status in postoperative patients with colorectal cancer in 1992. At week 2 and month 3 after operation, % change in lymphocyte count was significantly greater in the NYT group than the control group. Changes in T-cell number were also significantly greater in the NYT group than the control group at 3 and 6 months after operation. Percent change in PHA-stimulated lymphocyte proliferation was significantly greater in the NYT group than the control group at 6 months after operation. For interleukin-2 receptor-positive cell ratio 6 months after operation, there was a significant reduction from the preoperative value in the NYT group [18].

#### 3.1.2. Blood Diseases and Related Conditions

In 1999, Aoe et al. evaluated the efficacy of juzentaihoto or NYT combined with an erythropoietin (EPO) preparation in preoperative autologous blood donation for patients with cancer. The increase in reticulocyte count from the time of donation to the time of operation was larger in the group administered with juzentaihoto and NYT and the EPO group than the iron group. In addition, the increase in hemoglobin level was greater in the EPO group than the iron group and significantly larger in the juzentaihoto and NYT group than the EPO group [19].

In 1997, the same authors (Aoe et al.) reported the combined effect of EPO and NYT on anemia after autologous blood donation. Compared to the iron treatment group, the EPO and NYT group had significantly increased RBC count, hemoglobin, and hematocrit at the time of preoperative blood collection; this was not observed in the iron + EPO group [20].

In 1995, Yanagihori et al. revealed the efficacy of NYT for iron deficiency anemia due to menorrhagia. Elevation in hemoglobin levels from predosing to postdosing was significantly higher in the NYT group than in the control group. Meanwhile, palpitation, shortness of breath, and symptoms for which NYT should be effective (anorexia, night sweats, and cold limbs) were improved to a similar degree in the NYT and control groups [21].

In 2005, Motoo et al. reported that NYT ameliorates ribavirin-induced anemia in chronic hepatitis C. Maximum decrease in hemoglobin levels in the NYT group was significantly smaller than that in the control group [22].

#### 3.1.3. Symptoms Related to Otorhinolaryngology

In 1994, Miyazaki et al. reported the efficacy of NYT when used to improve xerostomia induced by oxybutynin hydrochloride. After a 2-week treatment with oxybutynin hydrochloride, 80% of patients developed xerostomia. NYT administration could reduce the symptom of dry mouth in 75% of patients. In addition, a reduction (0%) was not observed in the control group (nonadministration). Gum test also showed improvement in saliva secretion in the NYT group [23].

### 3.2. Use of Yasui's Classification Method to Categorize RCTs

**Figure 2:**
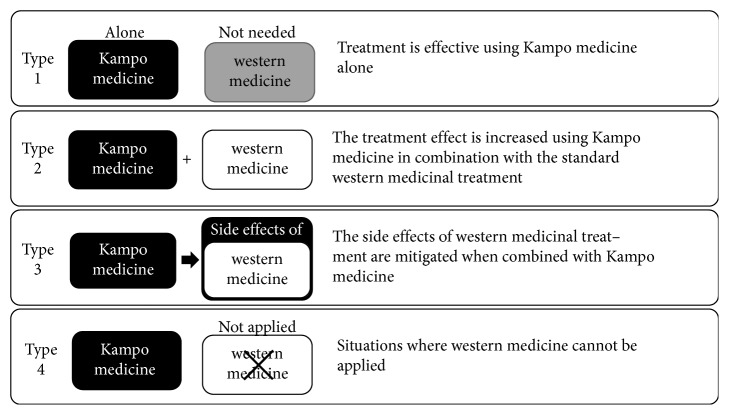
Number of randomized control trials using NYT as categorized using Yasui's classification method. Ten RCTs classified as Type 3 and 3 RCTs classified as Type 2.

Ten of the 13 RCTs were classified as Type 3, “the side effects of Western medicinal treatment are mitigated when combined with Kampo treatment” [11–18, 22, 23] and the remaining 3 were classified as Type 2, “the treatment effect of Kampo medicine is increased in combination with standard Western medicinal treatment” [19–21] (Figure 2).

## 4. Discussion

We reviewed RCTs where NYT was used to treat cancer and related conditions, refractory blood diseases and conditions, and otorhinolaryngologic symptom.

Yasui [4–10] reported his view regarding the use of Kampo medicine in modern medicine and suggested that the indications of Kampo medicine can be classified into 4 types: type 1, effective using Kampo medicine alone; type 2, effective combined with western medicine; type 3, mitigation for side effects of western medicine; and type 4, western medicine cannot be applied. In this study, we categorized the RCTs that we reviewed using Yasui's classification method (Figure 2). Ten of the 13 RCTs were classified as type 3 while the remaining 3 were classified as type 2; these correspond to side effects of treatment with western medicine and combination therapy using NYT and western medicine, respectively, and reflect the application of NYT in modern-day situations. In clinical trials, most studies were conducted using western medicine treatment, which prompted their classification into the type for combination therapy with western medicine and Kampo medicine. As a lack of effect using western medicine was observed in some cases, treatment with Kampo medicine can also be considered another possible application. Altogether, these reports suggest that treatment with NYT for pharyngeal vascular malformation [24], frailty [25], respiratory disorder [26], infectious disease [27], dysphagia [28], and sarcoidosis [29] may be feasible.

Many mechanisms of NYT have been reported in experimental studies, and the 12 components within NYT that contribute to these mechanisms have been summarized by Miyano et al [30]. These researchers evaluated the following effects of the 12 components: anti-inflammatory, antitumor, antioxidative, antiosteoporotic, antineurodegenerative, and neuroprotective activities; regulation of Na^+^/Ca^2+^ exchanger 3; inhibition of enzyme conversion from acyl ghrelin (active) to des-acyl ghrelin (inactive); maintenance of ghrelin levels as pachymic acid; elevation of adiponectin production; enhancement of skeletal muscle endurance; pain relief; and Ca^2+^ channel inhibition. Therefore, not only have these components been presented in the literature, but their effects (NYT formula) have also been elucidated. According to Makino et al. [31, 32], NYT improves Th1/Th2 balance, active oxygen scavenging action in inflammation, lupus nephritis, and anti-inflammation in mouse cytomegalovirus pneumonia; protects from candida infection; exhibits antitumor effects via natural killer activity, and enhances antitumor effect of anticancer drugs; suppresses lung metastasis of rectal cancer, enhances cancer vaccine through CD8 T cells; suppresses carcinogenesis; improves dementia via stimulating acetylcholine neuron; protects against mitochondrial dysfunction and intestinal mucosal failure which decline with age and necrosis of the skin flap; improves myelination hypoplasia by activating mitogen-activated protein kinase cascade, attenuates neuropathic pain (a side effect of anticancer drug treatment), recovers WBC, RBC, and PLT counts and olfactory disorder after anticancer treatment; mitigates transplant rejection; inhibits fibrosis and cirrhosis; and prevents hepatic disorder when sorafenib is administered. Their findings support the results of RCTs using NYT.

**Figure 3:**
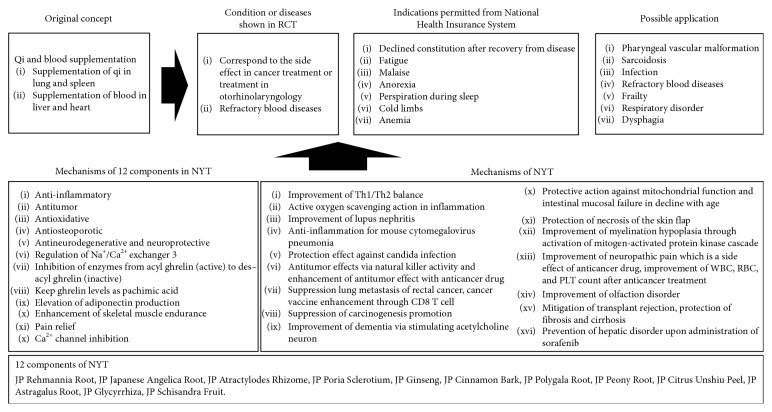
Overview of the original concept, indication, randomized control trials, mechanisms, and possible application of NYT in modern medicine.

Figure 3 conveys our understanding of the original concept, indication, RCT, mechanisms, and possible application of NYT in modern medicine. With regards to the original concept, NYT can be used to treat qi and blood deficiency pattern in the body, especially spleen and lung qi deficiency pattern and heart and liver blood deficiency pattern. These deficiencies are accompanied by symptoms such as fatigue, malaise, and cognitive impairment which are related to vital energy, hormone, and neurotransmitters; appetite loss and digestive disorder which are related to vital energy, neurotransmitters, and the autonomic nervous system; and body weight loss, mis-swallowing, dyspnea, and urinary disfunction, all of which are related to muscle weakness, sarcopenia, and the autonomic nervous system. Therefore, our review highlights the need to evaluate NYT for its side effects when used to treat cancer or xerostomia and refractory blood diseases. Based on the original concept, NYT can also be applied to a variety of other conditions and diseases such as frailty, dementia, or respiratory disorder.

Recently, NYT has been highlighted in the treatment of frailty and cognitive dysfunction in elderly patients. Although there were no RCTs related to this category, some surveys and open-label studies have been reported. Sakisaka et al. reported a cohort study of NYT with regard to frailty [33]; they showed that NYT improves grip strength in both hands and maintains muscle quality. Suzuki et al. conducted an open-label, noncomparative, prospective, multicenter, postmarketing survey of the safety and effectiveness of NYT in elderly patients [34]; they showed that NYT significantly improves visual analog scale scores for fatigue/malaise and anorexia and that it significantly decreases the proportion of patients expected to require nursing care, although adverse reactions were reported in 3.1% of patients, most of whom had gastrointestinal disorders (2.1%). Kudoh et al. conducted an open-label study on the effects of NYT on cognitive impairment and depression in patients with Alzheimer's disease with a two-year follow-up [35]. Their Mental State Examination (MMSE) results showed no significant differences between patients who received donepezil with NYT and those who received donepezil alone; however, significant improvement in the Alzheimer's Disease Assessment Scale‐cognitive component‐Japanese version score and the Neuropsychiatric Inventory (NPI) depression score were observed in patients who received donepezil with NYT. Ohsawa et al. also conducted an open-label study on the effects of NYT agaisnt anorexia, apathy, and cognitive dysfunction in frail patients with Alzheimer's disease [36]. They found that NYT caused a significant decrease in the score for anorexia and a significant increase in food intake by week 4. Moreover, the total NPI score and NPI subcategory scores for apathy and eating disturbance significantly decreased by week 4. A significant decrease in total caregiver distress scores was also observed from week 8. MMSE was significantly increased at week 12 compared to that at baseline. These reports suggest the possibility of NYT application for frailty and cognitive dysfunction in elderly patients.

With regards to side effects, some that are deemed adverse were reported in RCTs and a case report. Side effects of NYT were suspected in 3.6% of patients included in the RCTs. Symptoms exhibited by the patients include skin eruption, gastrointestinal disorder, fatigue, and liver dysfunction. A case report where skin eruption was diagnosed as a side effect of NYT treatment was reported by Ueda et al in 2001 [37]. A 73-year-old male complained of an itchy bullous eruption with erythema on the trunk, but he also had an erosion on the lower lip which appeared after he was given NYT. Patch testing to confirm these occurrences were NYT-triggered yielded a positive reaction and likewise, drug-induced lymphocyte stimulation test indicated a positive result. This suggests that physicians should pay keen attention to adverse effects when prescribing NYT.

## 5. Conclusions

By conducting RCTs, researchers could reveal the efficacy of NYT when used to treat refractory diseases and other conditions. With respect to modern medicine, many studies evaluated the additional effects that either occur by combining NYT and standard western medicinal treatment or handling the side effects of western medicinal treatment. In a future study, we aim to clarify the usefulness of NYT in modern medicine.

## Figures and Tables

**Table 1:** Overview of randomized control trials using NYT in this review.

Publication year	Author	Subjects	Intervention	Control	Outcome	Result	Adverse events
2005	Motoo et al.	Chronic hepatitis C patients treated with interferon alpha-2b and ribavirin (*n* = 23).	Interferon alpha-2b and ribavirin plus 9.0 g/day of Tsumura NYT (NYT group: *n* = 10).	Interferon alpha-2b and ribavirin (control group: *n* = 13).	Maximum decrease in RBC count (max∆RBC); maximum decrease in Hb level (max∆Hb); minimum Hb level (min Hb), WBC count, PLT count, T-helper 1 cell (Th1) count, T-helper 2 cell (Th2) count, Th1/Th2, and glutathione peroxidase level in peripheral blood.	Peripheral max∆Hb and min Hb were significantly improved in the NYT group (*P*=0.026 and *P*=0.079, respectively). No between-group differences were observed in max∆RBC, WBC count, PLT count, Th1 count, Th2 count, Th1/Th2, and glutathione peroxidase level. In addition, antiviral effects did not differ.	No adverse effect

2004	Oda et al.	Patients who underwent surgery for gynecologic cancer and received granulocyte colony-stimulating factor (G-CSF) for neutropenia during the first cycle of chemotherapy with cyclophosphamide, epirubicin, and cisplatin (total *n* = 8: ovarian cancer, 6; uterine cancer, 1; or fallopian tube cancer, 1).	Administration of 7.5 g/day of Kanebo NYT from 1 to 2 weeks prior to the start of the second cycle of chemotherapy (NYT group: *n* = 4).	Without NYT (control group: *n* = 4).	Nadir WBC and Neu count, the length of time for Neu count to fall below 1,000/*μ*L, total dose of G-CSF, duration of Neu counts under 1,000/*μ*L, and nadir Hb level and PLT count.	There was no significant difference between the groups in nadir WBC, Neu, and PLT counts or the length of time for the Neu count to fall below 1,000/*μ*L. Duration of Neu count under 1,000/*μ*L tended to be shorter in the NYT group than in control group during the second cycle, and became significantly shorter during the third cycle. Total dose of G-CSF tended to be lower in NYT group than in non-NYT group during the second cycle, and became significantly lower during the third cycle. Nadir Hb level during the second cycle, compared to that during the first cycle, was significantly shorter in NYT group, but not in the control group.	N/A

1999	Aoe et al.	Patients with gynecologic malignant tumors and preoperatively donated 800 mL or more of autologous blood. Intravenous administration of an iron preparation to patients with Hb level ≥14 g/dL. Randomization of patients with Hb < 14 g/dL to receive intravenous iron preparation + Kampo formulation + EPO or intravenous iron preparation + EPO. (total *n* = 90).	Intravenous administration of iron preparation (240 mg weekly) + intravenous drip infusion of 6000 units of EPO three times weekly + oral administration of 7.5 g/day of Tsumura Juzentaihoto or 7.5 g/day of NYT from the day of the first donation to the day before the operation (Kampo group: *n* = 36).	Intravenous administration of an iron preparation (240 mg weekly) from the day of the first donation to the day before the operation (iron group: *n* = 16); intravenous administration of an iron preparation (240 mg weekly) + intravenous drip infusion of 6000 units of EPO three times weekly, from the day of the first donation to the day before the operation (iron + Epo group: *n* = 38).	Hematological profile and serum biochemical profile measured before donation (before administration) and preoperatively (immediately after completion of administration).	The increase in reticulocyte count from the time of donation to the time of operation was larger in the Kampo group (*n* = 36) and EPO group than in the iron group (*n* = 15). The increase in Hb level was larger in the EPO group (1.73 ± 1.30 g/dL) than the iron group (0.92 ± 0.70 g/dL), and significantly (*P*=0.05) larger in the Kampo group (2.33 ± 1.11 g/dL) than the EPO group.	None

1997	Aoe et al.	Patients who donated 800 mL or more of blood for autologous transfusion. The control group (iron preparation only) consisted of patients who donated blood for autologous transfusion.	Iron preparation (intravenous administration of 80 mg three times a week) + Epogin (6000 units three times a week) + (9 g/day) of Tsumura NYT (NYT group: *n* = 26).	Iron preparation monotherapy (intravenous administration of 80 mg three times a week) (Iron group: *n* = 10); iron preparation (intravenous administration of 80 mg three times a week) + Epogin (6000 units three times a week) (iron + Epo froup: *n* = 37).	Hematological profile and serum biochemical profile measured before blood donation and before surgery.	Compared to iron group, but not iron + Epo group, NYT group had significantly increased RBC count, Hb, and hematocrit at the time of preoperative blood collection.	None

1995	Yanagihori et al.	Patients diagnosed with iron deficiency anemia (Hb, 9.0 mg/dL or less) due to menorrhagia and metrorrhagia associated with uterine myoma, uterine adenomyoma, endometrial polyp, etc. (*n* = 39).	5 g/day of Kanebo NYT + ferrous citrate 100 mg/day for 4 weeks (NYT group: *n* = 21).	Ferrous citrate 100 mg/day for 4 weeks (control group: *n* = 18)	Changes in hematological values, including serum iron and ferritin and subjective symptoms (general malaise, shortness of breath, and palpitation) from predose to postdose.	Elevation in Hb value from predoseto postdose was significantly higher in NYT group (*P*=0.01). Palpitation and shortness of breath and symptoms for which NYT should be effective (anorexia, night sweats, and cold limbs) were similarly improved in both groups.	None

1995	Okawa et al.	Patients with thoracoabdominal tumors undergoing radiotherapy (lung cancer: 42, esophageal cancer: 19, breast cancer: 9, rectal cancer: 7, cervical cancer: 33, and other cancers: 16) (total *n* = 116).	7.5 g/day of Kanebo NYT during radiotherapy (NYT group: *n* = 63).	Radiotherapy only (control group: *n* = 63).	Subjective symptoms (anorexia, general malaise, diarrhea, coldness, nausea, and vomiting), hematological parameters, body weight, and blood pressure. Biochemical values. Primary physicians evaluated the response of patients based on the above measures on a 4-point scale (marked, moderate, mild, or none).	There were no between-group differences in mean WBC count at baseline and at weeks 1–4. The proportion of patients with WBC count >3000/*μ*L at the end of treatment (weeks 4–8) was higher in NYT group (51/56) than in control group (42/60) (*P*=0.005). Improvement in subjective symptoms (at least mild response) was observed more frequently in NYT group (44/56) than control group (6/60) (*P*=0.0001). Improvement in laboratory test results (at least mild response) was observed more frequently in NYT group (43/56) than in control group (23/60) (*P*=0.0003).	4 patients in NYT group respectively had drug eruption, abdominal discomfort, abdominal pain + diarrhea, and diarrhea.

1995	Sugimachi	Postoperative patients with stage I–IV gastric cancer undergoing gross curative resection (*n* = 46).	Fluoropyrimidine anticancer drug + 7.5 g/day of Kanebo NYT from 2 to 14 weeks postoperatively (NYT group: *n* = 27).	Fluoropyrimidine anticancer drug alone (control group: *n* = 19).	Hematological measures, body weight, PS, subjective symptoms (appetite, nausea/vomiting, and diarrhea) at 14 weeks after the start of administration.	Change in body weight, PS: no significant difference between the groups. Decrease in RBC count, platelet count: a smaller decrease in NYT group, although not significantly smaller. Decrease in WBC count: no significance between the group difference. Degree of improvement in subjective symptoms: no significance between the group difference.	None

1994	Miyazaki et al.	Patients who complained of dry mouth out of 20 patients who were diagnosed with psychogenic frequency or unstable bladder (chronic cystitis, neurogenic bladder) and received oxybutynin hydrochloride (6 mg/day) for 2 weeks (*n* = 16).	18.0 g/day of NYT under oxybutynin hydrochloride medication for 4 weeks (NYT group: *n* = 8).	Oxybutynin hydrochloride alone for 4 weeks (control group: *n* = 8).	Severity of dry mouth (on a 5-point scale), chewing gum test, frequency of urination evaluated by interview at baseline, 2 weeks, and 4 weeks.	After a 2-week treatment with oxybutynin hydrochloride, 16 of 20 patients (80%) developed xerostomia symptoms. NYT administration for 2 weeks could reduce the symptom of dry mouth in 75% patients, but 0% in control group. Gum test also showed improvement of saliva in NYT group.	N/A

1994	Yamamoto et al.	Patients undergoing cancer chemotherapy or radiotherapy (*n* = 40).	Chemotherapy + 7.5 g/day of Kanebo NYT (chemo with NYT: *n* = 11) Radiotherapy + 7.5 g/day of Kanebo NYT (rad with NYT: *n* = 10).	Chemotherapy + cepharanthine 2 tablets t.i.d. (chemo in control: *n* = 12). radiotherapy + cepharanthine 2 tablets t.i.d. (rad in control: *n* = 7).	Four performance status items evaluated on a 5-point scale, nausea/vomiting evaluated on a 4-point scale, hematology, and urinalysis.	NYT treatment significantly improved myelosuppressive symptoms but not subjective and objective symptoms associated with anticancer drug administration. It also improved anorexia and fatigue/malaise during radiotherapy.	One patient had acute hepatitis with unknown causal relationship with NYT.

1994	Hasegawa et al.	Patients with ovarian, uterine cervical, or uterine corpus cancer undergoing cyclophosphamide, adriamycin, and cisplatin therapy. (*n* = 32).	7.5 g/day of Kanebo NYT and 7.5 g/day of Kanebo Juzentaihoto for 5 weeks from 1 week before to 4 weeks after administration of anticancer drugs (*n* = 19).	No administration (*n* = 13)	Pretreatment and posttreatment myelosuppression and nephrotoxicity evaluated by hematology, and subjective symptoms (general malaise, anorexia, and vomiting) evaluated on a 4-point scale using a standard questionnaire.	Kampo medicine treatment did not significantly affect decreases in WBC, RBC, and PLT counts but tended to promote their reversal. Kampo medicine also reduced nephrotoxicity (i.e., normalized blood urea nitrogen (BUN) level and reduced creatinine fluctuation). Subjective gastrointestinal symptoms were not improved.	No adverse effect

1993	Mizuno et al.	Gynecologic cancer (uterine cervical, uterine corpus, ovarian, etc.); more than 1 month since the completion of the initial treatment or treatment for recurrence; at least one of the following subjective symptoms: anorexia, fatigue/malaise, decreased physical strength, cold limbs, night sweats, and lightheadedness.	7.5 g/day of Kanebo NYT for 12 weeks (NYT group: *n* = 46).	No NYT administration (control group: *n* = 44).	Improvement in subjective symptom scores was used to measure efficacy.	Subjective symptoms such as physical strength, general fatigue, coldness in hand and foot, and night sweating were significantly improved in the NYT group compared to the control group. The efficacy determined by the doctor according to symptom improvement and side effects was significantly higher in the NYT group than in the control group.	Only one case showed appetite loss and general fatigue which was categorized as a side effect.

1993	Ohara et al.	Patients with cancer including gastric cancer (*n* = 91), colorectal cancer (*n* = 63), breast cancer (*n* = 18), and other cancers (*n* = 6) receiving an anticancer drug (tegafur 400 mg/day or 600 mg/day) (total *n* = 178).	7.5 g/day of Kanebo Hochuekkito for 6 months (*n* = 57) or 7.5 g/day of Kanebo NYT for 6 months (NYT group: *n* = 56).	Tegafur alone for 6 months (control group: *n* = 49).	Subjective symptoms (appetite, nausea/vomiting, etc.), objective symptoms (performance status (PS), body weight, blood pressure, etc.), hematology (blood counts, carcinoembryonic antigen, and immunosuppressive acidic protein), and biochemistry at baseline and after 2, 4, and 6 months of treatment.	Subjective symptoms compared between predose and postdose, nausea/vomiting, bowel movement abnormality, motivation, and fatigue/malaise were significantly improved in NYT group. Overall, improvement was noted in 19/56 patients (33.9%) in NYT group, and 7/49 patients (14.3%) in control group, with significant differences in the percentage of patients showing improvement between the groups. Overall, objective symptom improvement was noted in 22/56 patients (39.3%) in NYT group and 10/49 patients (20.4%) in control group, with significant differences in the percentage of patients showing improvement between NYT group and control group. Hematology: there were no significant differences between the groups. Cancer type: only in patients with gastric cancer, the percentage showing improvement in both subjective and objective symptoms was significantly greater in the NYT group than control group.	Side effects were observed in 11.5%.

1992	Araki et al.	Postoperative patients with colorectal cancer on chemotherapy (*n* = 23).	9.0 g/day of Tsumura NYT from the start of postoperative oral feeding (NYT group: *n* = 12).	No treatment (control group: *n* = 11).	Peripheral blood cell count, percentage of T cells (%), phytohemagglutinin (PHA) lymphocyte transformation, lymphocyte surface markers (CD4, CD8, and CD25), NK cell activity (%), and interleukin-2 responsiveness, measured preoperatively, and at postoperative week 2 and months 3 and 6 as indices of immunological status. Patient prognosis (observation for 3 years and 6 months to 4 years and 4 months) in both arms. Prognostic nutritional index.	Percent change in lymphocyte count: greater in arm 1 than arm 2 at week 2 and month 3 after operation (*P*=0.05). Change in the T cell number (in %): Greater in arm 2 than arm 1 (*P*=0.05) at week 2 after operation, but greater in arm 1 than arm 2 (*P*=0.05) at months 3 and 6 after operation. Percent change in PHA-stimulated lymphocyte proliferation: Greater in arm 1 than arm 2 at month 6 after operation (*P*=0.05). Changes in NK cell activity (in %) and prognostic nutritional index: no significant difference between arms. Changes in the number of CD4-and CD8-positive cells (in %) and interleukin-2 responsiveness ratio: all tended to be greater in arm 1 than arm 2 at week 2 and month 3 after operation. Interleukin-2 receptor-positive cell ratio: Tended to be greater in arm 1 than arm 2 at week 2 and month 6 after operation. At month 6 after operation, there was a significant reduction from the preoperative value in arm 1 (*P*=0.05).	N/A

WBC: white blood cell; Neu: neutrophil;RBC: red blood cell; PLT: platelet; PS: performance status; N/A: not assigned.
